# Effectiveness of music therapy with relaxation technique on stress management as measured by perceived stress scale: Retraction

**DOI:** 10.1097/MD.0000000000019635

**Published:** 2020-03-13

The article, “Effectiveness of music therapy with relaxation technique on stress management as measured by perceived stress scale”,^[1]^ which appears in Volume 98, Issue 15 of *Medicine*, is being retracted over concerns regarding the validity of the statistical analysis. Authors were unable to provide the original dataset to address the concerns raised.
